# Oral Rehabilitation Using Noninvasive Restorative Approach for Late Mixed Dentition of Preterm Birth Child with Amelogenesis Imperfecta

**DOI:** 10.1155/2020/8816835

**Published:** 2020-11-21

**Authors:** Abdulfatah Alazmah

**Affiliations:** Department of Preventive Dental Science, College of Dentistry, Prince Sattam University, Alkharj 11942, Saudi Arabia

## Abstract

Preterm birth children comprise about 6% of live births around the world. It is known that premature children exhibit oral anomalies that could affect the function and/or appearance of their dentition in addition to their medical needs. A diagnosis of amelogenesis imperfecta (AI) can present a challenge for both the patient and the treating clinician. This can be more complicated in the case of child treatment, where cooperation and some of the treatment modalities for adults can not be considered. Conventional management of such children is not possible due to the ongoing process of growth and development and the ability of the child to cope with the extensive and lengthy treatment procedure. This article highlights a minimally invasive method for managing AI using adhesive and full-coverage restoration that requires no tooth preparation; this allows the structural integrity of the teeth to be maintained, along with their vitality. As a result, the child will have teeth with better function and aesthetic, to improve eating, appearance, and self-confidence.

## 1. Introduction

According to the World Health Organization, preterm birth is defined as when the baby is born before 37 weeks and stays alive. It is a common condition affecting about 15 million babies around the world yearly and may lead to death. Preterm babies are divided, depending on the weeks of gestational age. Babies born earlier than 28 weeks are considered severely preterm, while the moderate and late preterm categories are given to infants born between the weeks 28 to 32 and 32 and 36 weeks, respectively [1].

At the molecular level, the formation of tooth enamel involves many highly controlled and varied processes. The developmental pathways of normal biomineralisation could be affected by the environmental conditions leading to process disturbing [2]. Amelogenesis imperfecta (AI) is a type of developmental defect of enamel (DDE) that represents a group of developmental conditions that are genomic in origin and that affect the structure and clinical appearance of enamel of all or nearly all the teeth in a more or less equal manner; this may be associated with morphologic or biochemical changes elsewhere in the body. The prevalence of amelogenesis imperfecta differs depending on the geographical region; 1 : 700 to 1 : 14000 is reported in the United States [3]. However, considering the needs for AI prevalence study across Saudi Arabia, the enamel defect generally reaches a prevalence of around 75% [4]. The main subtypes of AI were classified by Carl Witkop depending on the phenotype, inheritance mode, and perceived pathogenesis to hypoplastic, hypocalcified, hypomaturation, and hypomaturation-hypoplastic, and these groups were later divided into further subtypes. The disturbance of the ameloblasts was considered the reason for the hypoplastic AI type, where the formation cells fail to generate the enamel matrix properly, leading to reduced thickness or amount of the enamel [3]. On the other hand, hypocalcified AI is caused by the inability of crystallites to properly nucleate, leading to a decreased mineral content. Abnormal cleavage of the enamel matrix or proteinase activity abnormality during the maturation stage that results from abnormal processing of the matrix protein is responsible for the hypomature type [5]. It can be difficult to diagnose if the enamel is hypomineralised or hypoplastic and to establish how the appearance, colour, and position of defects may affect the quality of the enamel. Therefore, factors should be considered prior to the formulation of a treatment plan for AI patients, including the age of the patient, defect type and severity, the extent of the extraction, and the patient's socioeconomic status. In addition, patients' needs and demands should be considered, including relieving symptoms (like sensitivity) and aesthetic considerations [6].

The importance of early diagnosis and management for children affected by AI is essential. Once the chief complaint is resolved, an intermediate management plan should be developed to aim for long-term survival of the tooth and to preserve the teeth until permanent restorations can be placed, often a few years later. On the other hand, children's ability to cope with the treatment will play a significant part in the decision-making process, especially for difficult and long-term restorative needs [7]. As definitive management may not occur for AI children until they reach adulthood, the least invasive approach necessary to meet the aesthetic demands of each individual should be adopted [8].

## 2. Case Report

**Figure 1:**
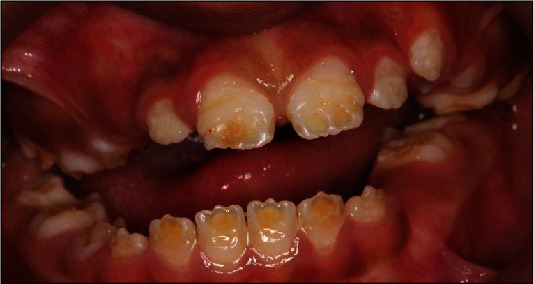
Frontal view with teeth in occlusion.

**Figure 2:**
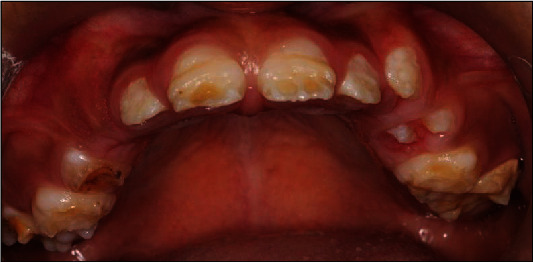
Upper occlusal arch.

**Figure 3:**
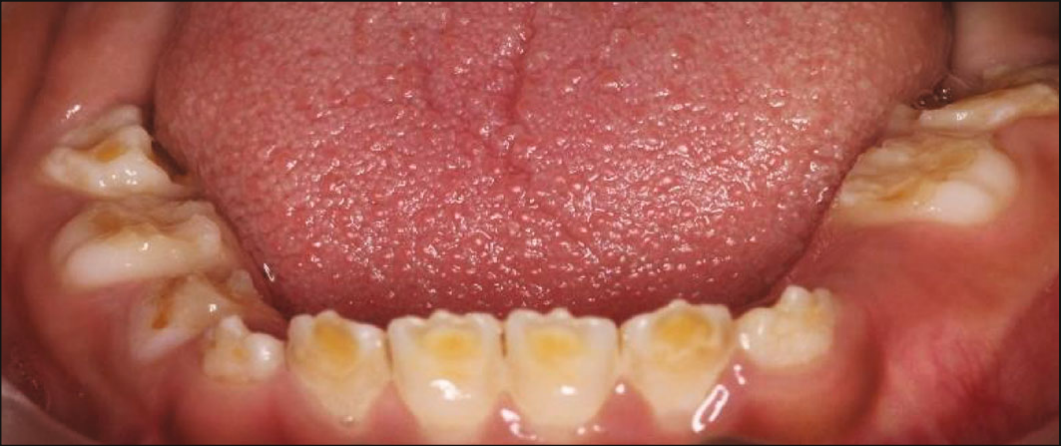
Lower occlusal arch.

**Figure 4:**
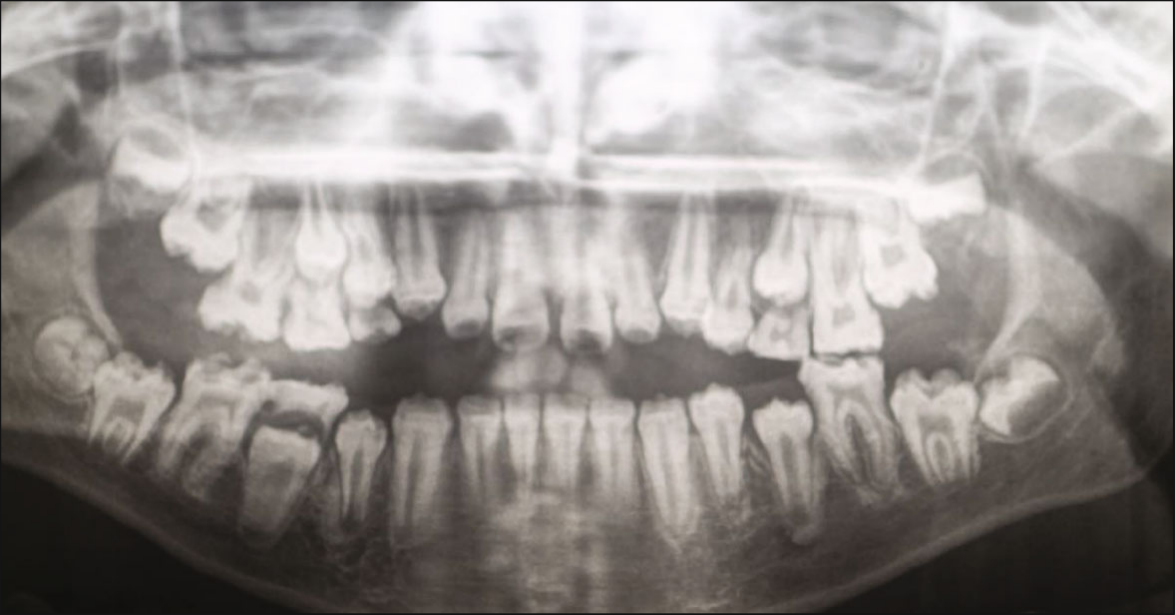
Dental panoramic tomogram (DPT).

A 10-year-old girl attended with her father after she was referred to Paediatric Dentistry Clinic for diagnosis and treatment. The main chief complaint was the discoloured front teeth. Furthermore, bullying at school was occurring due to her teeth appearance. In addition, general teeth sensitivity was affecting the upper central incisor, especially during brushing and temperature change. A detailed dental and medical history was obtained from the child as well as the parents. Dental family history revealed that the relatives did not suffer from similar dental conditions. However, the patient was born prematurely at 8 weeks, and she was in an incubator for the first 8 weeks of birth. This was followed by a good recovery leading to not requiring any medication or hospitalisation during her first few years after her birth. Upon intraoral examination, we noted a mixed dentition stage, with partially erupted teeth, and caries-free primary and permanent teeth (except for the mobile upper right first primary molar). Regarding the occlusion, we noted a class II division one incisor relationship, skeletal class II relation with increased vertical proportion, midline shift to the left side, and an 8 mm anterior open bite, and the child was occluding solely on the left first permanent molar. The hypoplastic phenotype was affecting the enamel of both dentitions (more prominent in permanent dentition) with demarcated yellowish-brown discolouration at the incisal third of lower incisors, upper central incisors, first molars, and primary teeth. The features obtained from both clinical and radiographic examination were consistent with a possible diagnosis of hypoplastic AI (Figures 1–3). A dental panoramic tomogram (DPT) taken showed all permanent teeth and hypoplastic defects affecting the teeth, including the unerupted second molars (Figure 4).

The treatment planning was based on preserving the remaining tooth structure until the patient can reach the age when she can receive a definitive treatment, keeping in mind the need to relieve the symptoms including sensitivity, improve the occlusion, and aesthetic demands. Following discussion with the child and parents, it was agreed to use a noninvasive approach with no tooth structure removal. The treatment plan was divided into a prevention, restorative, and maintenance phase. In the prevention phase, enforcement of oral hygiene instructions was done at every visit. Sodium fluoride varnish 22,600 ppm (Duraphat, Colgate, USA) was applied to reduce dental sensitivity for three consecutive weeks to reduce teeth sensitivity, protect and prevent remaining structures from future loss, and, at the same time, to avoid caries development [9, 10].

During the planning visit, orthodontic consultation was obtained. Due to the skeletal open bite tendency of the patient combined with class II and the increased vertical proportion, the orthodontic treatment may include skeletal anchorage device or orthognathic surgery. It was recommended to delay the orthodontic treatment and reevaluate the case after one year, so a referral to Orthodontic department for periodic follow-up until treatment is initiated was done.

**Figure 5:**
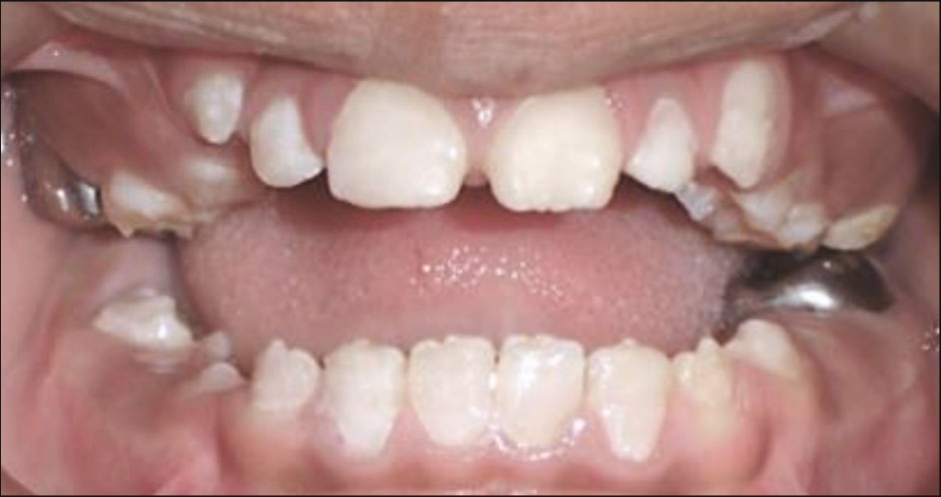
Intraoral front.

**Figure 6:**
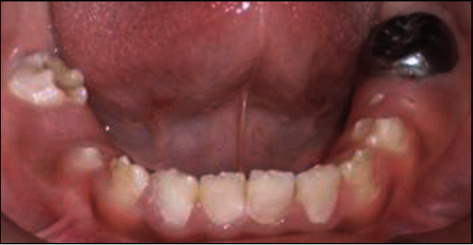
Intraoral lower occlusal.

Restorative treatment was done for the permanent incisors and first molars. A direct composite veneer was performed to the upper and lower incisors: following the manufacturer's instruction, an adhesive system was applied (Optibond Solo plus, single component, Kerr Co., CA, USA). The enamel shade was applied in the cervical region, and shade A2 and shade A1 were used to the middle and cervical thirds (GRADIA® Anterior, GC corporation, USA) Finishing and polishing procedures of the restorations were performed using sequential Sof-Lex discs (3M ESPE, Seefeld, Germany). Regarding the first molars, the upper right and lower left were affected more by posteruptive breakdown when compared to the other two molars. As a result, a preformed metal crown (stainless steel crowns; 3MTM ESPETM, St Paul, MN, USA) was done and cemented using glass ionomer cement (Aquacem®; Dentsply, Milford, DE, USA). The other two molars were restored by composite, as the remaining enamel was sufficient to perform a retentive filling (Figures 5 and 6).

**Figure 7:**
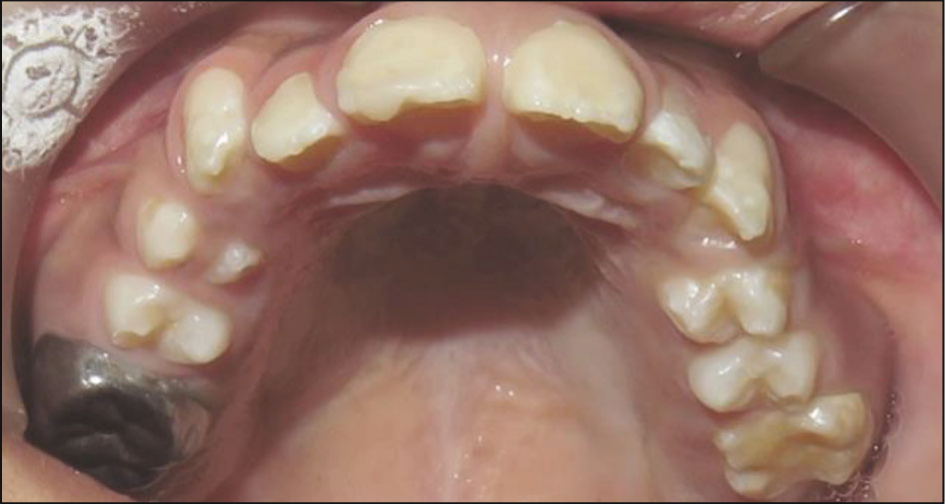
Intraoral upper occlusal.

Follow-up was done for 15 months. The treatment showed a maintained tooth structure, good oral hygiene, and eruption of premolars (Figure 7).

## 3. Discussion

The complications of a preterm birth may affect both the general and dental health of the patient. The risk of developing an infection, respiratory distress syndrome, bleeding intraventricularly, and ductus arteriosus are among the common conditions related to preterm birth [11]. Dentally, complications may affect the oral structure, like high-arched palate and alveolar ridge notching [12]. Regarding the teeth shape and size, it has been reported that permanent first molars and incisors crown to tooth ratio may be reduced [13]. Moreover, DDE can affect the enamel of both primary and permanent teeth like amelogenesis imperfect, and conditions can affect the permanent teeth, but only molar-incisor hypomineralisation has been linked to preterm births [14].

Enamel defects can affect children and adolescents in a variety of ways, including aesthetics, function, and psychosocial impacts. Eliminating dental sensitivity and enhancing patient smile are part of the aims of the treatment, where colour adjustment is the most important factor. The issue of teasing was also evident. Improvement is important for psychosocial well-being, particularly during the mixed and early permanent dentitions. Chronological hypoplasia was considered as a differential diagnosis as no family history of teeth abnormalities was reported. However, a diagnosis of AI was made based on the fact that all the teeth were affected and associated with a skeletal anterior open bite [15].

A recent systematic review looking for the association between DDE and preterm birth children concluded the following: “In conclusion, the results of this meta-analysis showed a strong association between DDE and preterm birth.” Furthermore, it highlighted the importance of early diagnosis and establishing prevention, in order to prevent both short- and long-term complications as preterm children are four times at higher risk to develop DDE [16].

The management of patients with enamel defects is a lifelong process. Regular reviews are needed to monitor restorations. It is important to maintain the patient's cooperation with oral hygiene instructions to ensure long-term durability of these restorations. Monitoring permanent teeth eruption and reported symptoms are crucial.

Class II malocclusion represents the most common skeletal discrepancy, and the class II/1 incisal relationship was found in association with a range of vertical skeletal patterns [17]. The minimal invasive restorative management approach leads to an improvement of patient eating, speaking, and aesthetic problems. However, this will not ultimately be achieved until this treatment is later completed by a multidisciplinary treatment approach which is aimed at managing the patient's malocclusion including the anterior open bite.

## 4. Conclusion

It can be very difficult for practitioners to decide how best to restore newly erupted permanent teeth that have hypomineralised or hypoplastic enamel. Keeping in mind that limited child cooperation may sometimes increase the need for pharmacological management approaches like sedation, focusing on the best interim treatment by practitioners in the most conservative way will be more accepted by the children and their caregivers.
